# Upright radiotherapy for breast cancer: a pre-clinical study considering photon and proton beam access, plus arm positioning

**DOI:** 10.3389/fonc.2025.1668109

**Published:** 2025-12-10

**Authors:** Gordon Sands, Chung Tin Lo, Jemma Nunn, Mark Ramtohul, Simon Ingram, David Cobben, Elizabeth Chabner, Tracy Underwood

**Affiliations:** 1 Research Department, Leo Cancer Care Ltd, Crawley, United Kingdom; 2 Department of Medical Physics and Biomedical Engineering, University College London, London, United Kingdom; 3 Radiotherapy Department, University Hospital Birmingham, Birmingham, United Kingdom; 4 Radiotherapy Department, Clatterbridge Cancer Centre, Liverpool, United Kingdom; 5 Masthead, Chabner XRT, Port Chester, NY, United States

**Keywords:** upright, radiotherapy, breast, treatment planning, Chabner XRT Bra

## Abstract

**Introduction:**

Upright radiotherapy has gained increasing attention in recent years due to its potential advantages, including lower room costs, improved patient comfort, and possible anatomical/physiological benefits. In this pre-clinical study, we assess the feasibility of implementing upright radiotherapy for breast cancer by evaluating beam access, inframammary skin fold size, field length, and set-up comfort across a range of upright positions.

**Materials and methods:**

Twenty-one healthy participants were enrolled in the study. Each participant was set-up on an upright patient positioning system (Eve from Leo Cancer Care Ltd) with three different arm positions (arms up, arms down, and arms behind). Setups were conducted both without a bra (topless) and with the Chabner XRT Bra for Radiotherapy, an indexable bra designed for immobilisation during treatment. Optical surface scans were acquired, and the external contour of the breast was used to approximate a clinical target volume. Beam access was evaluated in multiple regions of the breast while field size and the ISF were measured for the different positions. Participants rated their comfort using a survey. ArUco markers were employed to evaluate ease of setup by measuring the unaided reproducibility of each upright position.

**Results:**

The ISF was smallest in the arms-up position when participants wore the Chabner XRT Bra. Beam access for photon treatment planning was assessed for 15 participants. Arm position significantly affected photon beam angle flexibility; with arms down, participants had fewer available angles for beam angle entry. The Chabner XRT Bra consistently reduced the required field length across all positions. Participants could typically reposition themselves with sub-centimetre accuracy, without any assistance from the study team.

**Conclusion:**

Overall, this study demonstrates the feasibility of delivering breast radiotherapy in the upright position for both photon and proton treatments. The arms-up position was preferable in terms of photon beam access. While photon beam access was limited for the arms down position, it remained achievable in the majority of cases. The use of the Chabner XRT Bra significantly reduced the size of the ISF, which may help lower skin toxicity, as well as the field length required for treatment, potentially decreasing unwanted lung dose.

## 1 Introduction

Over recent years, there has been renewed interest in positioning patients who require radiotherapy upright, on a robotic chair, so that they may be rotated through a fixed treatment beam. So-called “upright radiotherapy” stands to reduce the equipment costs associated with rotating, gantry-based beam delivery systems. The gantry-less concept should also reduce shielding costs and volumes: as the treatment beam is fixed, primary radiation shielding is only required in a single direction. for photons and protons (1).

Multiple upright CT scanners and patient positioning systems are entering the commercial market (2). In addition to these products, “in-house” chair-based systems have been used for decades in various proton and ion clinics around the world (3). McCarroll et al. describe the installation of a custom upright chair within a conventional rotating gantry room for photon radiotherapy for those who could not tolerate supine positioning (4). It has been reported that upright body positioning can offer scope to improve both psychological and physiological comfort, for example, for patients requiring radiotherapy for head and neck, prostate, and breast cancers. There is also limited evidence to suggest that treating the patient in an upright position may bring anatomical/dosimetric benefits to certain tumour sites (such as prostate and lung) (5, 6). However, if upright systems are to enter routine clinical use, optimal treatment positions, immobilisation methods, and supine/upright anatomical changes must first be investigated in pre-clinical studies.

Globally, breast cancer is the most common clinical indication requiring radiotherapy (7). One 2003 study estimated the optimal radiotherapy utilisation rate for breast carcinoma to be 81%, but found actual global utilisation rates to vary between 24% and 71% (8). Another paper from 2016 estimated that in Brazil, only 40% of patients with breast cancer had access to radiotherapy (9). Reducing the cost of photon radiotherapy treatment rooms through gantry-less systems could play an important role in improving the global accessibility of this vital treatment. The conventional setup for breast radiotherapy usually involves a supine breast board where the patient stretches their arms above their head, to give access to the breast. This body position is known to be problematic for some patients, particularly those who come to radiotherapy with pre-existing upper limb mobility issues, either stemming from previous surgical treatment (10, 11).

In this work, we build upon a previous study by Boisbouvier & Underwood et al. (12), which assessed several factors associated with breast radiotherapy in the upright position. These factors included patient comfort, measures of positional reproducibility, and the impact of bras specifically designed as immobilisation accessories for radiotherapy (the commercially available Chabner XRT Bra and the prototype Support4All bra). Overall, the small cohort of patients considered by Boisbouvier & Underwood et al. viewed upright body positioning favourably, with patients largely preferring the upright position they tested over the supine position used for their treatment. Historically, for breast radiotherapy, large inframammary skin folds have been associated with increased skin toxicity (13). The Boisbouvier & Underwood et al. paper reported that specialised bras were highly effective in reducing/eliminating inframammary skin fold (ISF) measurements for upright body positions.

One potential issue raised by Boisbouvier & Underwood et al. was photon beam access for upright radiotherapy of the breast and whether, because breast tissue lies more medially for upright body positions, the contralateral breast could start to be clipped by the treatment fields and receive unwanted dose. If that were to be the case, not only a radiotherapy bra but also additional immobilisation would be required to keep the contralateral breast out of photon treatment fields. This is a topic we investigate in the present study.

Further, while Boisbouvier & Underwood et al. started to investigate both arms-up and arms-down treatment positions in terms of comfort, they did not consider how radiotherapy field size (and potential lung exposure) was likely to vary with arm position, with and without a bra. Here, we investigate likely treatment field size and inframammary skin fold (ISF) measurements for three different upright body positions: arms up, arms by side and arms back. We also investigate the range of beam angles that could be applied for a photon-based treatment plan for arms up and arms back, considering that photon beams must not clip the ipsilateral arm/arm support or the contralateral breast. Building on Boisbouvier & Underwood’s successful utilisation of specialised bras with upright body positioning, we here utilised the Chabner XRT Bra. Inframammary skin fold measurements were considered alongside the ease of set-up reproducibility (defined as the precision with which participants were able to reposition themselves without external assistance) and the comfort of healthy volunteers.

## 2 Material and methods

### 2.1 Participant demographics and upright positioning

After approval from the Local Ethics Committee for the Department of Medical Physics and Biomedical Engineering at University College London (Project ID 26179/001), 21 healthy female volunteers were recruited, aged from 24 to 83. They gave informed consent to participate in the study. Recruitment was weighted towards volunteers with large bra cup sizes (challenging anatomies for radiotherapy of the breast). The participants’ median bra size as measured by the research team was 36DD, with the maximum size being 30H (cup sizes ranged from B-H; bra band sizes ranged from 30”-42”). The women were aged 24–83 years (mean 58.8 years). They had heights of 1.55 m- 1.75 m (mean 1.65m). Body Mass Index (BMI) values were 20-42 (mean 26.7).

For this study, each participant was fitted with a specialised bra designed as an immobilisation accessory for radiotherapy: the Chabner XRT Bra (available commercially). Participants were measured around the upper chest (above the bust), across the fullest part of the bust, and below the bust to select an initial Chabner XRT Bra size, which was then verified through a fitting process. The Chabner XRT Bra fitting process prioritised lifting the left breast to minimise, as much as possible, the left inframammary skin fold (ISF), under the assumption of left-sided radiotherapy treatment.

The participants were then positioned on a demonstration version of the Eve upright patient positioner/radiotherapy chair (Leo Cancer Care). This device was ergonomically matched to the complete Eve system. However, it was designed to be transportable; it had more manual controls, but did not translate/rotate. Following the recommendation from Boisbouvier & Underwood et al., the seat pan was tipped down 15 degrees from horizontal, and the backrest was tilted backwards by 10°for all trials in this study. Compared to a true seated position, this slightly “perched” body position was used to reduce the extent to which the abdomen bunched against the breasts, particularly for women with a large abdominal pannus.

The following arm positions were trialed: (1) arms above head, using physical arm supports custom-designed for use with the Eve upright patient positioner and its regular, rectangular backrest; (2) arms along the body, placed next to the participant’s sides, (3) arms behind the body, using a custom-designed, narrower backrest (with cutaway sections for the arms, armrests and hand-grips behind the body). The various setups and the number of setups are shown in **Table 1**.

**Table 1:** Summary of positioning tests for each participant.

Bra on or topless	(1) Arms above head	(2) Arms along body	(3) Arms behind body	(4) Left arm up, right arm along body
Without any bra (topless)	3 repeat set-ups	3 repeat set-ups	3 repeat set-ups	3 repeat set-ups
Wearing the Chabner XRT Bra	3 repeat set-ups, with the Chabner XRT Bra removed between each set-up	3 repeat set-ups, with the Chabner XRT Bra kept on forall	3 repeat set-ups, with the Chabner XRT Bra kept on for all	3 repeat set-ups, with the Chabner XRT Bra kept on for all

Between the repeats, the participants were asked to stand up, stretch/move their body freely, and then attempt to reinstall themselves in the same position as before.

For each arm position, inframammary skin fold (ISF) measurements were made for each breast using a tape measure, both with the participant topless and wearing the Chabner XRT Bra, A paired sample t-test was used to compare the means of the ISF in the various scenarios.

### 2.2 Anatomical markers for radiotherapy field boundaries and positional reproducibility measurements

While each participant stood topless with their arms down, their suprasternal notch (SSN) was palpated, and a cross was marked on their skin at this level to serve as a reference point for the treatment planning analysis. This marker was subsequently used to define the superior border of treatment fields.

To quantify the intra-participant set-up reproducibility, a verified algorithm capable of automatically detecting QR-like ArUco markers (14), was employed in conjunction with two perpendicular cameras on fixed tripods. The cameras were positioned approximately 2.5 meters from the left breast, one facing the participant from the front and the other from the left-hand side. A set of 10 ArUco markers, each measuring exactly 3 cm x 3 cm, were printed and securely taped to each participant’s skin to ensure consistent marker positioning across repeated trials using the upright patient positioning system (**Supplementary Figure 1**). Seven of these ArUco markers positions were visible within the anterior camera’s field of view, located at the following anatomical landmarks: the supra-sternal notch; the tip of the xiphoid process; approximately 2cm above the xiphoid tip; left nipple; right nipple; approximately 2cm below the lowest point of the right breast; near the superior bony prominence of the Angle of Louis, underneath the clavicle, above the right breast). The remaining three ArUco markers were visible to the left lateral camera and placed at: the left axilla, left medial breast tissue (approximately halfway between the nipple and the left lateral field border), and the left lateral field border. The position of the markers can be seen in **Supplementary Figure 1**. Exact anatomical placement of the markers was not required; rather, the markers were positioned at a range of locations relevant to breast radiotherapy positioning and marker positions were kept consistent (via tape) for each individual participant.

### 2.3 Optical scans for photon treatment planning considerations

3D optical scans of participants’ body surfaces were taken using an Intel D415 depth camera and the itSeez3d v2.0 software. For each scan, a researcher walked around the participant in a complete circle, slowly translating the camera up and down to capture the complete body surface, excluding the head but including the chin to ensure it wasn’t near the treatment area.

The resulting 3D scans were imported into Autodesk Meshmixer 3.5.474; in this software, the sternal notch (reference point for the superior border of the treatment field, marked in pen for each participant) was made into a 3D marker on the skin’s surface. The STL files were imported into 3D Slicer 5.2.2 and converted into synthetic CT dicom files using a bulk density assignment for the entire body contour. These files were imported into Raystation v. 2023b. Photon plans and beams were then created for different participants and body positions, with and without the Chabner XRT Bra.

A conventional approach for photon beam borders was adopted to analyse beam access, using reference points from the external anatomy. The details of how external contours were used in this process are included in the **Supplementary Data**. Following the generation of an estimated clinical target volume (CTV), a photon treatment plan was created with the isocentre positioned at the centre of the CTV. To evaluate beam access, three slices from the pseudoCT dataset were analysed: *upper slice*, located 2cm inferior to the superior border of the treatment volume; *middle slice*, corresponding to the midpoint of the treatment volume as defined by the Treatment Planning System (TPS); *lower slice* located 2cm superior to the most inferior slice of the breast.

For each of these three breast slices, the available range of entrance beam angles for photon beams was assessed. Entrance beam angles were excluded if they met any of the following criteria (a) intersected the arm, (b) intersected the contralateral breast, (c) entered through the backrest or (d) entered through the physical arm support. Exit dose through the backrest or arm supports was not considered a limiting factor in this analysis.

The optimal beam access angle was defined as the angle at which the beam created a non-divergent border along the edge of the CTV. No margin was included on the field. This approach is conceptually similar to a half-beam block technique, but instead uses the multileaf collimators (MLCs) and beam angle selection to achieve the non-divergent edge along the midline of the field. Further information on the method used to simulate treatment planning can be found in **Supplementary Data**. The field length (in the cranio-caudal direction) was also measured, as this serves as a surrogate marker for the extent of unwanted lung irradiation during breast treatment.

### 2.4 Participant comfort

**Figure 1:**
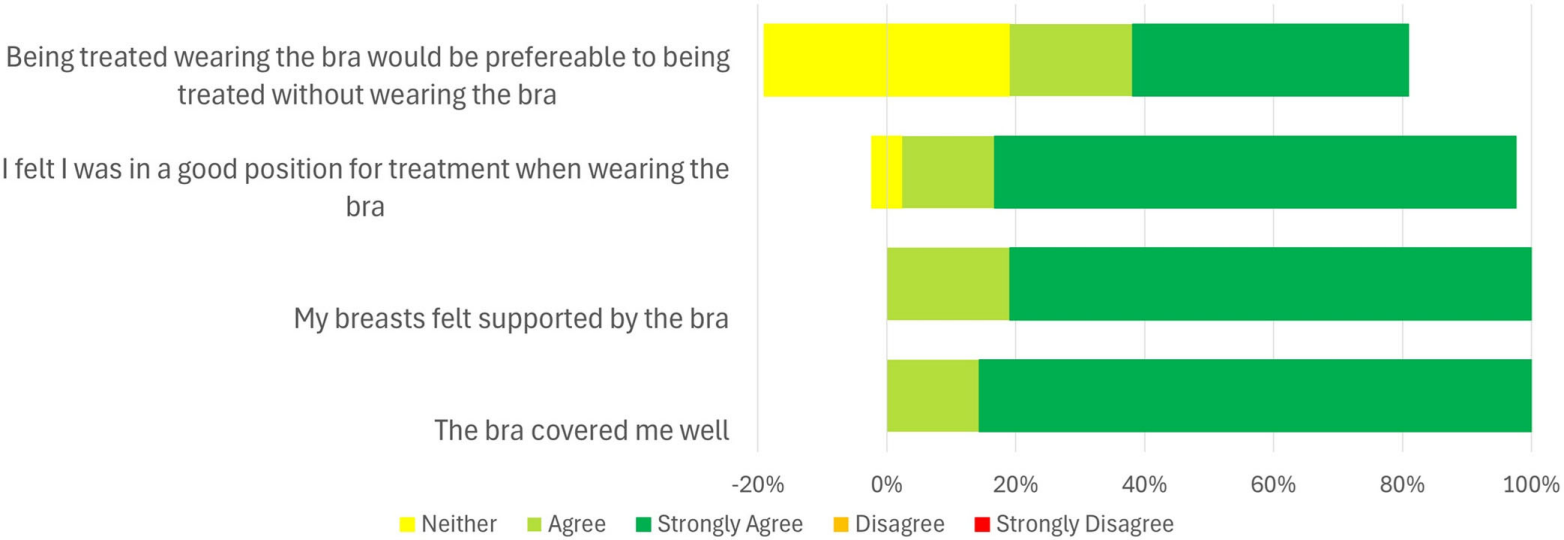
Results from the comfort questionnaire on the Chabner XRT Bra (n=21).

**Figure 2:**
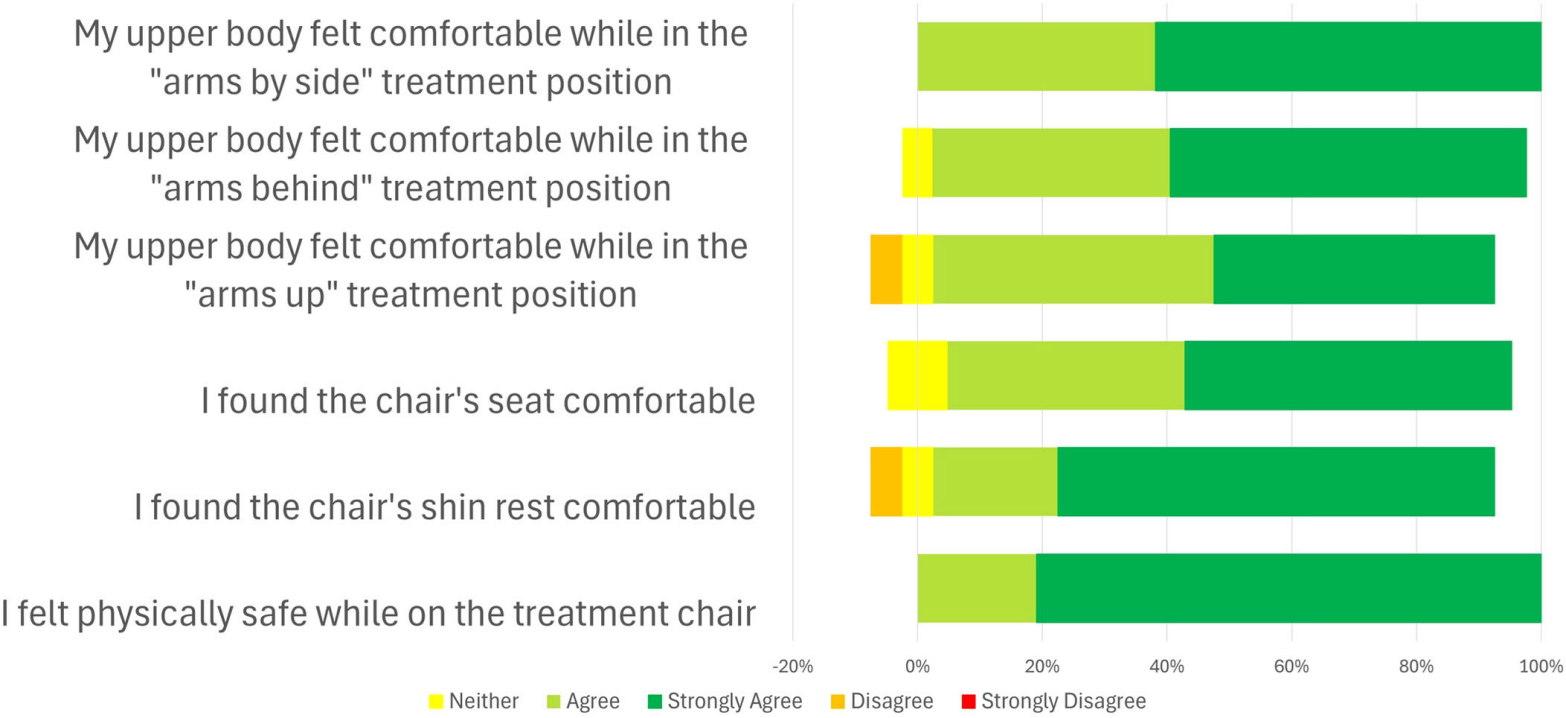
Results from the comfort questionnaire on the Leo Cancer Care upright patient positioning system (Eve) (n=21).

Participant comfort levels in various postures and with the Chabner XRT Bra were assessed using a five-point Likert scale questionnaire (Strongly Agree, Agree, Neutral, Disagree, Strongly Disagree). The specific questions are presented in **Figures 1**, **2** and **Supplementary Figure 4**. This questionnaire was specifically developed for the purposes of this study.

## 3 Results

### 3.1 Arm positioning

**Figure 3:**
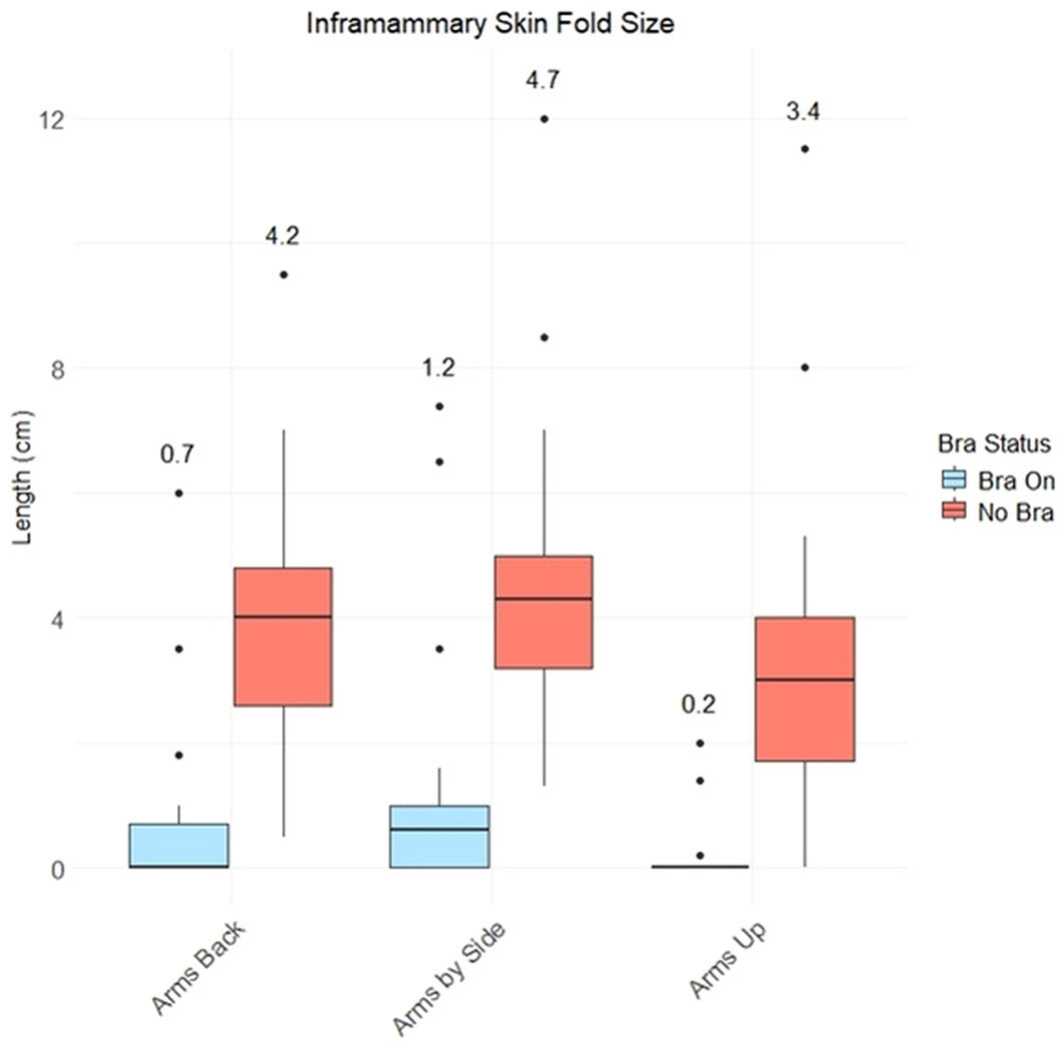
Left breast inframammary skin fold (ISF) measurements for different upright body positions, with and without the Chabner XRT Bra. The mean ISF value is written above each boxplot.

ISF values are expected to be more substantial for upright body positioning as the breast tissue falls medially under gravity (compared to supine positioning, where it falls laterally). **Figure 3** plots left breast ISF measurements for all participants. As expected, the Chabner XRT Bra substantially lifts the breast and reduces the ISF (mean ISF values for the scenarios considered are included in **Figure 3**). Using a paired t-test and a significance level of p=0.05, statistically significant differences were found between the ISF measurements with and without the Chabner XRT Bra, for each of the three body positions (“arms up, “arms by side” and “arms back”). Using the same statistical testing methods, without The Chabner XRT Bra, ISF measurements were found to vary significantly between body positions, with “arms up” positioning resulting in the lowest ISF, followed by “arms back” and then “arms by side” positioning. When the Chabner XRT Bra was added, however, ISFs only differed at the p=0.05 level between the “arms up” and “arms by side” body positions. Of the 63 measurements (21 participants, 3 arm positions) made with the Chabner XRT Bra on, the ISF was measured as 0cm in 38 cases (60.3%). Without the Chabner XRT Bra, there was only one participant and position that had a 0cm ISF, this was in the arms up position.

### 3.2 Photon beam access and treatment planning data

Of the 21 participants, 15 were included in the photon beam access component of the study. Five participants were excluded due to technical issues with the surface scan reconstruction. One participant was excluded due to a pre-existing arm mobility issue, which meant they could not raise their arms sufficiently to compare photon beam access for arms-up and arms-back treatment positions. Arms-by-side positioning was not considered for this beam access element of the study. While arms-by-side positioning would likely be appropriate for upright treatments using en-face proton fields (15), it is incompatible with the lateral beam access is required for photon radiotherapy. One-arm-up positioning was also not considered in this part of the study: photon beam access would likely be comparable to the two-arm-up scenario. The chin was never close to the treatment area as the participants had their heads back against the backrest and their chins raised.

#### 3.2.1 Length of treatment field required to cover the breast

**Figure 4:**
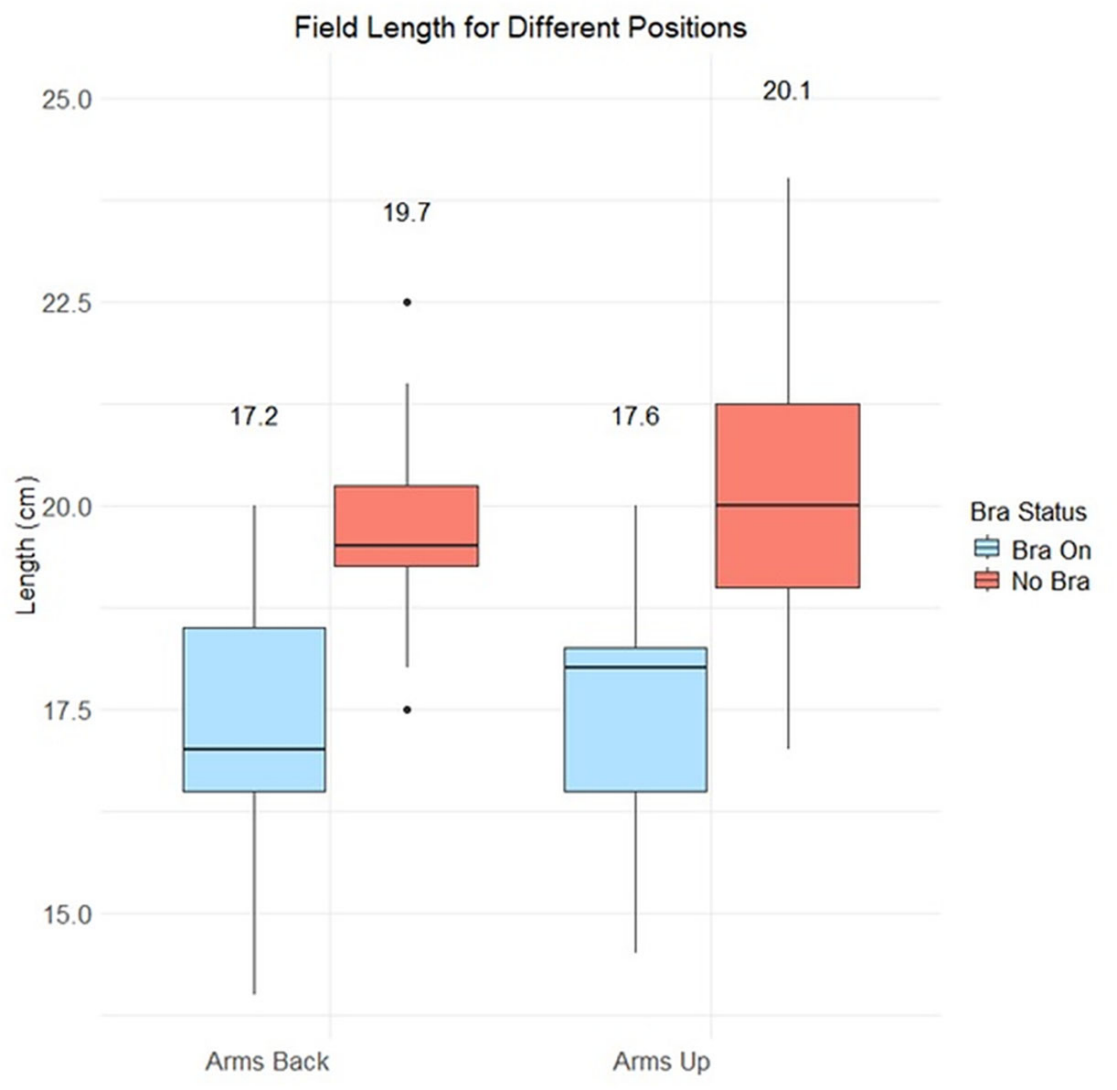
Comparison of field lengths in the treatment planning system, for different setups. Mean values are written above each boxplot.

**Figure 4** presents a box plot of the superior-inferior length of the photon treatment fields required to cover the breast. For both arm positions, introducing the Chabner XRT Bra led to a statistically significant reduction in mean length of the field (paired t-test, p<0.01 for both arms-up and arms-back positions) The mean reduction was 2.5cm for both setups, with a median reduction of 2.7cm arms-back and 2.1cm in the arms-up position). Without the Chabner XRT Bra, the mean difference in field size between the arms-up and arms-back positions was 0.4cm, which was not statistically significant (p=0.13). With the Chabner XRT Bra, the mean difference in field size between the arms-up and arms-back positions was also 0.4cm (p= 0.16). The field length will impact the volume of lung treated and the impact of the imaging field size.

#### 3.2.2 Optimal photon beam angles

In conventional supine photon radiotherapy for breast cancer, a few different planning approaches are employed. While use of CTV-based VMAT is growing, the traditional method involves paired anterior oblique and posterior oblique beams, to cover the whole breast. These angles are typically used with partially closed (half-beam-blocked) jaws, which create non-divergent beams along the back border of the target, reducing non-therapeutic dose by creating a sharp dose fall off for the lung. This technique can be further refined with MLC-based modulation or field-in-field methods. In this study, for simplicity, conventional anterior and posterior oblique pairs of beams were used to represent typical photon beam access requirements.

**Figure 5:**
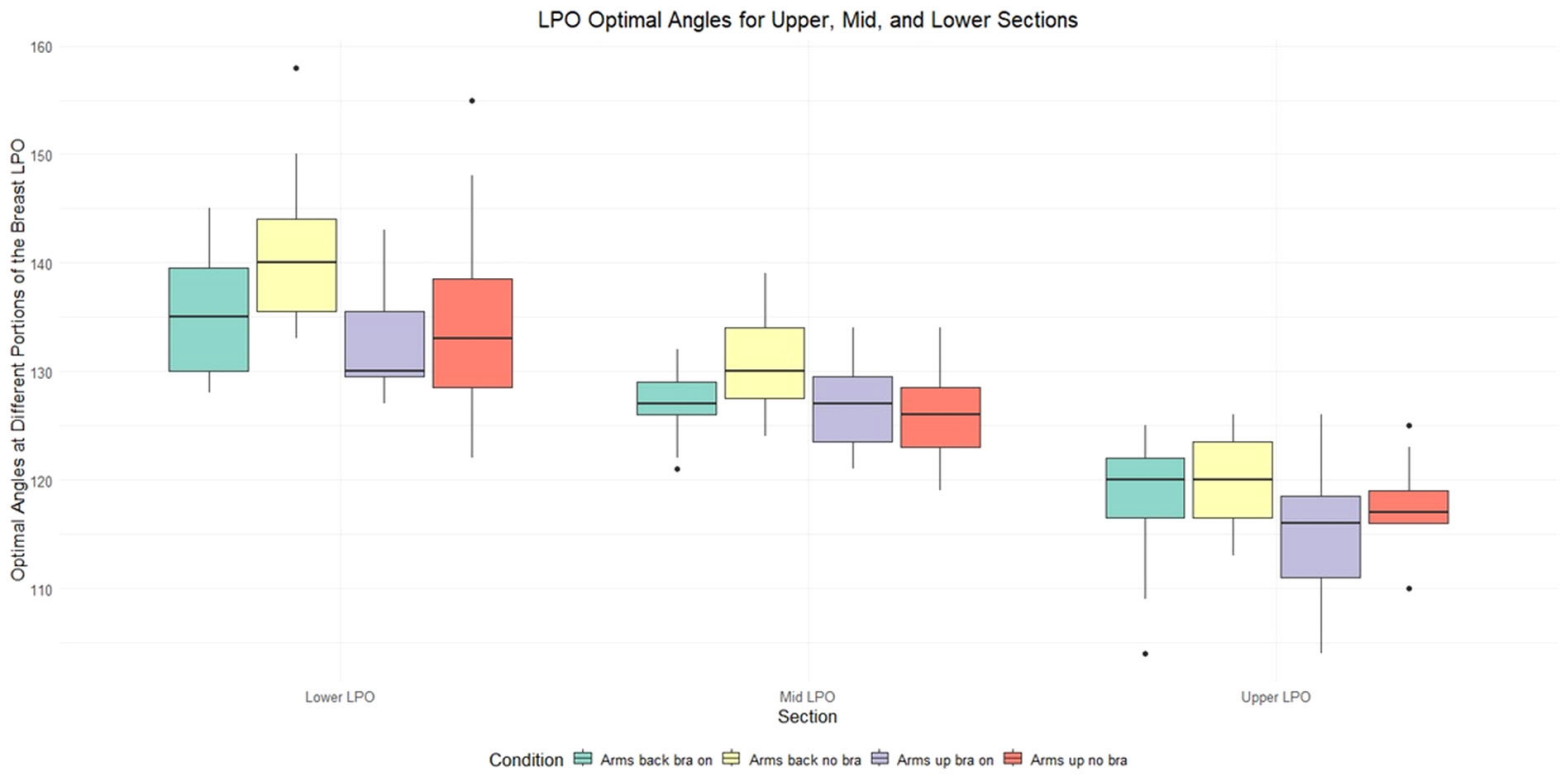
The optimal Left Posterior Oblique (LPO) photon beam angles for different sections of the breast (lower, mid and upper sections), for various upright set-ups: arms back Chabner XRT Bra on (green), arms back no bra (yellow), arms up Chabner XRT Bra on (purple), arms up no Bra (red). The data highlights that different beam angles become optimal for different superior-inferior sections of the breast. While the scans were obtained in the upright position, they were flipped to Head First Supine (HFS) to match the orientation seen in supine radiotherapy.

Optimal photon beam angles were defined by drawing a straight line from the breast tissue’s midline edge to the lateral edge of the breast tissue, as visualised on the left surface of each participant’s 3D body scan. This leads to an angle where the divergence of the beam is matched for both fields. This should lead to all the breast tissue being treated while minimising excess dose spillage. **Figure 5** shows the optimal angles for the three superior-inferior breast sections (as defined in section 2.3) across the 15 participants. These beam angles follow the head first supine convention, where 0 degrees indicates the participant is directly facing the beam delivery system.

**Figure 5** indicates that, with upright positioning, different beam angles become optimal for different superior-inferior breast regions. This could be implemented using a sliding window, slow-rotating gantry, and sliding jaw technique. While this may sound similar to a standard VMAT treatment, there is a subtle difference: at each beam angle, the jaw would move, covering only a small section of the breast as the MLC covers the breast tissue at that angle. As a result, each region of the breast effectively maintains a localised half-beam block, which shifts along the superior-inferior axis. The optimal beam angles are shown in **Figure 5** for the LPObeam, the RAO beam exhibited a similar trend.

#### 3.2.3 Available beam angles

For each of the 15 participants considered for this photon treatment planning section of the study, 4 treatment plans were created: (1) arms-up Chabner XRT Bra on, (2) arms-up no bra, (3) arms-back Chabner XRT Bra on, (4) arms-back no bra. Across the 60 treatment plans, 12 were unsuccessful due to beam-access issues. Of these, ten were in the arms-back position, and two were in the arms-up position. All unsuccessful plans were due to the ipsilateral arm obstructing the desired beam path (arm clipping), although this was sometimes combined with other factors. Arm clipping was most prominent in the upper 2cm section of the breast. The 2 unsuccessful arms-up plans (with and without the Chabner XRT Bra) were both for the same participant, who was unable to raise their arms sufficiently high without discomfort (likely due to frailty, rather than a specific arm mobility issue). For 5 participants, the arms-back position proved insufficient, with arm clipping interfering with targeting the upper section of breast. However, for all 15 participants, at least one of the arm positions (arms-up or arms-back) allowed for adequate beam access for the photon plans. In general, the arms up position offered greater flexibility, particularly for the upper breast, due to reduced risk of arm clipping.

**Figure 6:**
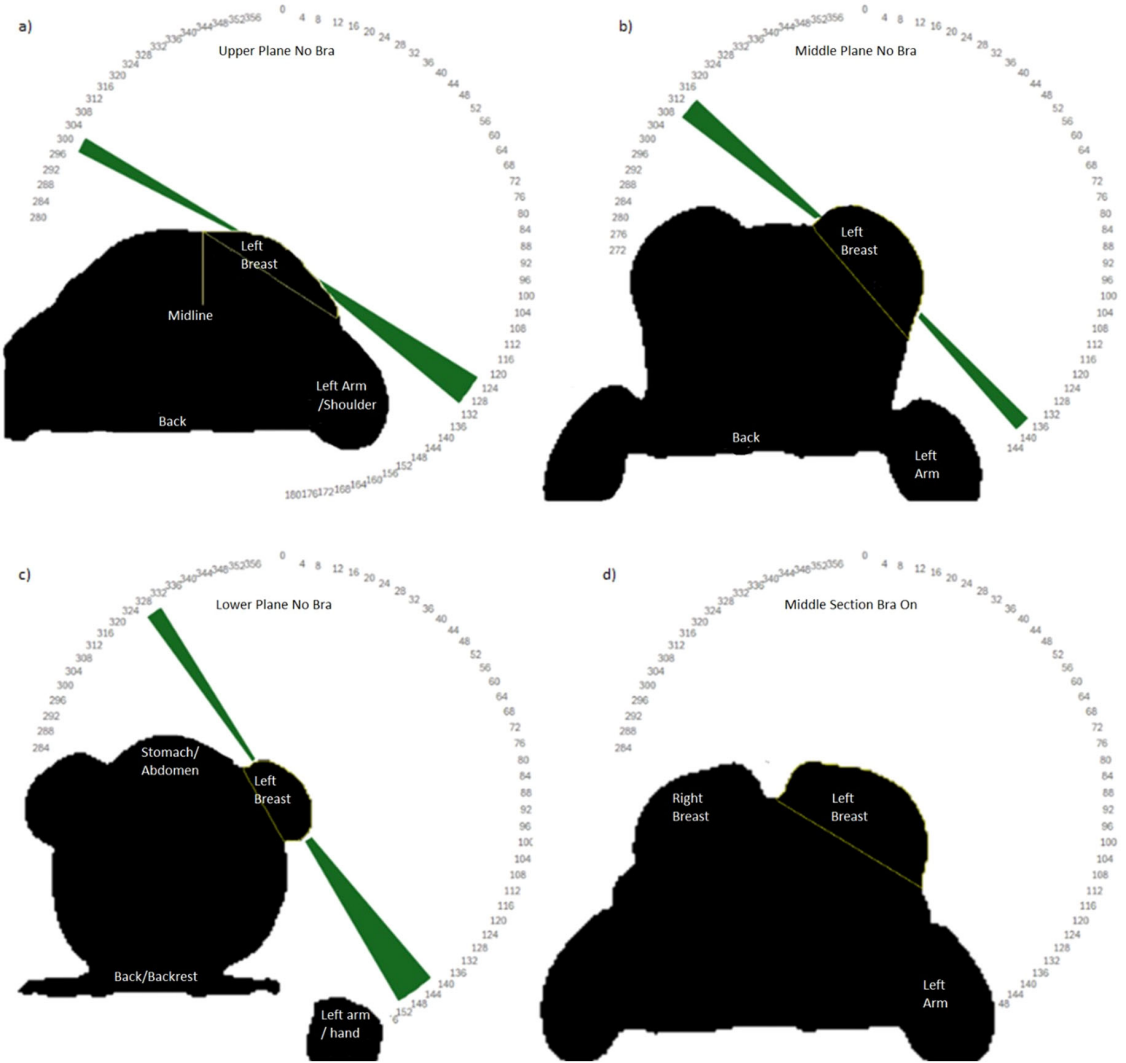
Slices through the 3D surface scans for a single participant in the upright arms-back position. Top-bottom, left-right **(a)** upper plane Chabner XRT bra off **(b)** mid plane Chabner XRT bra off, **(c)** lower plane Chabner XRT bra off, **(d)** mid-section Chabner XRT Bra on. The range of angles that would be suitable for photon treatment fields is shown in green. In setup 4, there are no available angles due to contralateral breast clipping combined with arm clipping.

**Figure 6** demonstrates photon beam access constraints for a single participant in the upright arms-back position. The image shows four single planes through the 3D surface scans. This figure highlights that different photon beam angles are needed to cover different superior-inferior sections of the breast for this upright, arms-back position. No cases were found where a single beam angle could treat the whole breast in the arms-back position. For the upright, arms-up position, 19 of the 30 scenarios could be treated using just a single angle. However, these were not always optimal, i.e. the angle of entry and exit did not coincide with the curvature of the breast.

**Figure 7:**
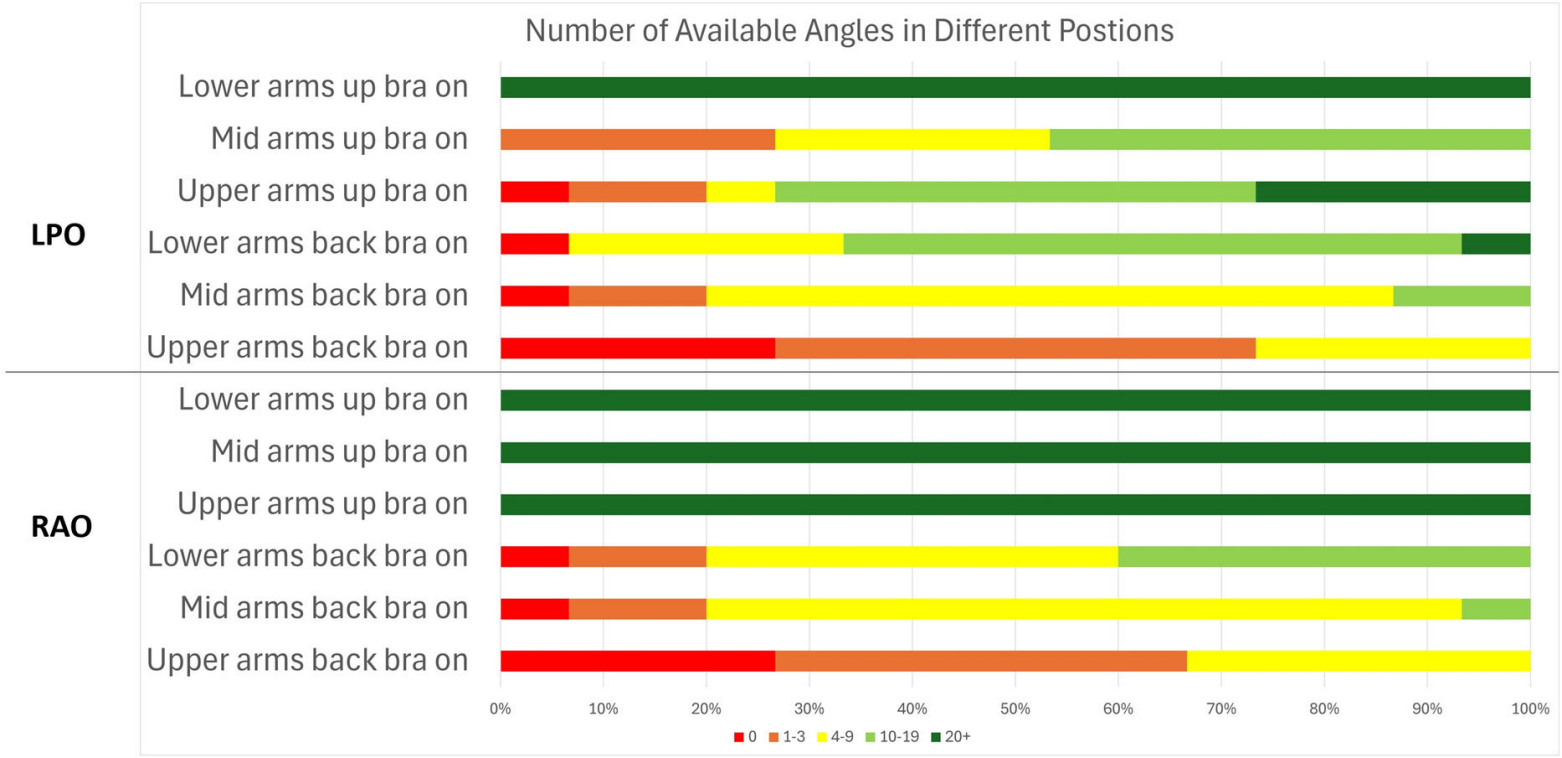
The number of available photon beam angles for each segment of the breast (upper, mid and lower) and body position setup.

**Figure 8:**
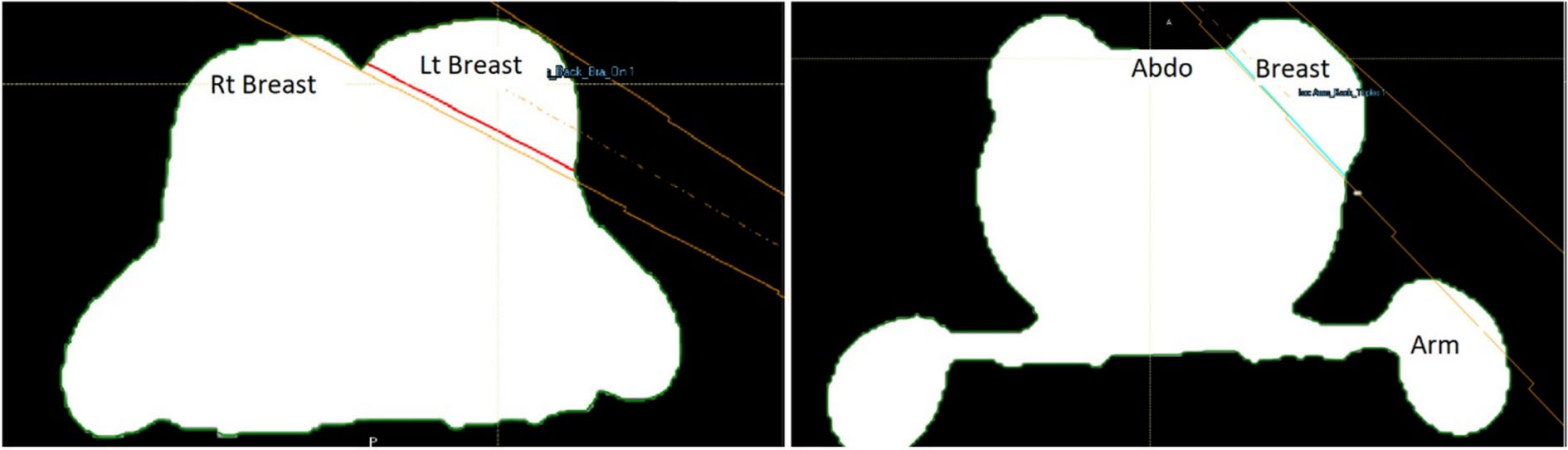
A single plane of the 3d model with labelled regions to indicate beam clipping. The left panel **(a)** shows how the contralateral breast could be clipped. The left and right breast are indicated in the image. The lines show the beam. The right panel, **(b)** shows how, following the curve of the breast, the ipsilateral arm could be clipped. The arm, breast tissue and abdominal tissue are indicated. The lines show the beam.

Contralateral breast clipping reduced the number of available beam angles for all 15 participants in both positions, as it was one of the criteria used to define beam angles as “available.” The number of available angles for each setup can be seen in **Figure 7**. However, on its own, it did not cause any planning attempt to fail. For the arms back position, contralateral breast clipping combined with arm clipping led to one unsuccessful planning attempt in the middle breast section for a case where the Chabner XRT Bra was used. This case is shown in **Figure 8a**.

**Figure 8b** shows an example of arm clipping for the arms back position. While shifting the angle of the beam could allow treatment of the breast, it would result in increased unwanted dose to organs at risk, such as the lung and heart. **Table 2** summarises the number of successful plans and where the failures occurs.

**Table 2:** Considering the possibility/otherwise of applying photon fields which cover the breast without clipping (i) the arm (entrance and exit), (ii) the backrest (entrance clipping only), (iii) the contralateral breast (entrance and exit), or (iv) large sections of the abdomen, for different superior-inferior sections of the breast (upper, mid and lower).

Description of plans and sections analysed	Arms up chabner XRT bra on	Arms up topless	Arms back chabner XRT bra on	Arms back topless	Total
Total Number of plans	15	15	15	15	**60**
Number of unsuccessful plans	1	1	5	5	**12**
Upper section slice failures	1	1	4	3	**9**
Mid-section slice failures	0	0	1	3	**4**
Lower section slice failures	0	0	1	0	**1**

Bold indicates total.

### 3.3 Unaided participant reproducibility of setup

**Figure 9:**
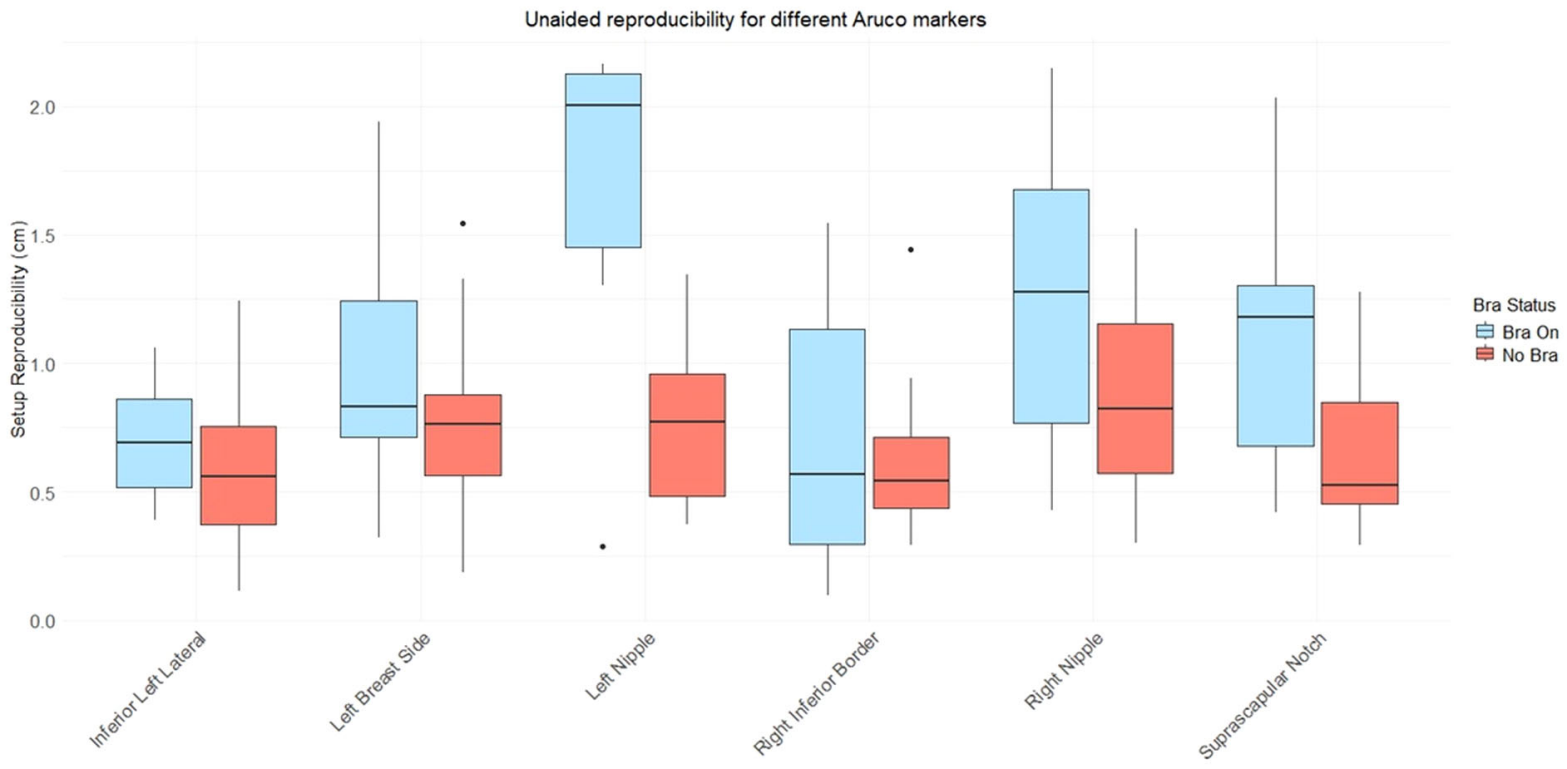
Boxplot showing mean displacement values for individual ArUco markers for all participants, for the arms up position. Participants reinstalled themselves in the arms up position three times, unaided by the study team. The mean displacement was calculated for each participant, from the differences between these three repeated setups.

A subset of the ArUco markers were used to assess participants’ “unaided” reproducibility, that is how accurately participants were able to reinstall themselves in the same upright treatment position (without help or intervention from a radiographer or other member of the study team) after standing up and moving/stretching their bodies. **Figure 9** considers this reproducibility for the arms-up position. The boxplots represent the displacement between markers after the participant (1) stood up from the chair, (2) took the Chabner XRT Bra off and put it back on as relevant (for the subset of data where the Chabner XRT Bra was considered), and (3) sat themselves back down, attempting to replicate their previous body position without assistance from the study team. Set-ups were repeated 3 times, and geometric displacements between markers were considered between repeat 1 & 2, repeat 2 & 3 and between repeat 1 & 3, before a mean was taken across these three pairs of repeats. For ArUco markers fixed on skin in the breast region but outside of the Chabner XRT Bra, the mean unaided reproducibility values across all participants ranged between 0.57cm (No Bra Inferior Left Lat Marker) and 1.08cm (Bra on, Suprascuplar Notch Marker), indicating that participants could typically position themselves with sub-centimetre accuracy. The largest mean variation was for the left nipple with the Chabner XRT Bra on, this rose to 1.65cm, compared to 0.75cm with the Bra off. For ArUco markers fixed on skin that sat within the translucent Chabner XRT Bras, in particular the nipples, the unaided reproducibility measures worsened when the Chabner XRT Bra was used, suggesting that the nipples did not always return to the same position within the Chabner XRT Bra when the participant took it off and put it on again. This effect was particularly evident for the left nipple, perhaps because lifting the left breast was prioritised (assuming a left-sided treatment).

**Figure 10:**
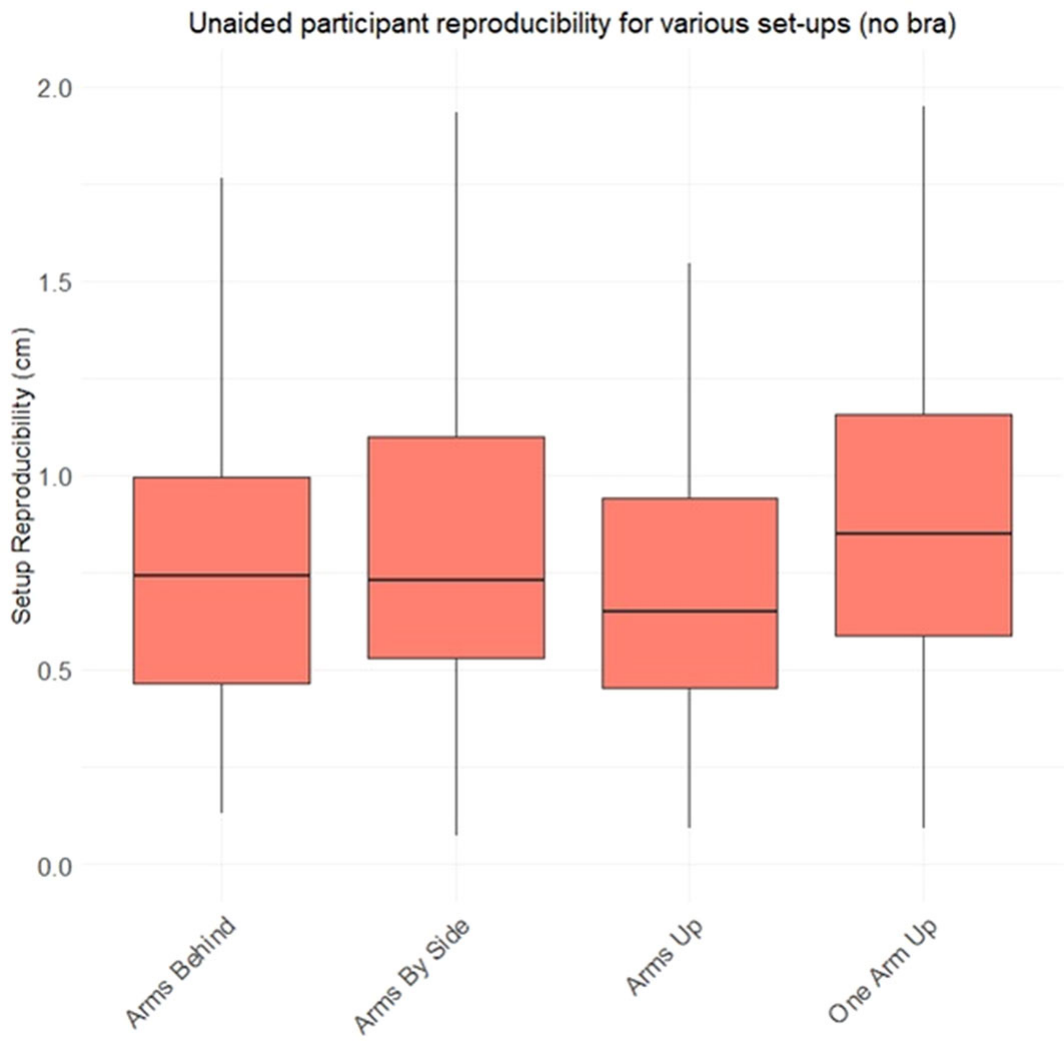
Boxplot showing average ArUco marker displacement between set-ups, across all markers and all participants. Participants reinstalled themselves in position three times, unaided by the study team. In this figure, only set-ups without the Chabner XRT Bra are considered. For one arm up, only 17 participants took part.

**Figure 10** combines topless data for all available ArUco markers to consider variation in unaided participant reproducibility between the different poses. The date indicates relatively little variation in unaided reproducibility between the different poses. The only statistically significant difference found was between the arms up and one arm up position (p=0.006 using a wilcoxon rank sum test).

### 3.4 Comfort questionnaires

Using questionnaires, both the comfort of the Chabner XRT Bra and the upright position were assessed. Overall, participant feedback on both was positive.

#### 3.4.1 Bra comfort

**Figure 1** summarises the responses to questions on Chabner XRT Bra comfort. The healthy participants responded positively to the Chabner XRT Bra, with thirteen out of the twenty-one (62%) saying they would prefer to be treated with the Chabner XRT Bra on, and the remaining eight (38%) responding neutrally. When asked about whether they felt pressure with the Chabner XRT Bra on, only two respondents (9.5%) reported pressure or discomfort while it was being put on. The complete questions and responses are included in the **Supplementary Data**, **Supplementary Figure S4**.

#### 3.4.2 Patient positioning system comfort

**Figure 2** shows the responses to questions on the comfort of the patient positioning system across a variety of treatment positions. In general, participants found the various set-ups to be comfortable. However, one participant (4.8%) found the arms-up position uncomfortable, and another (4.8%) found the shin rest uncomfortable.

## 4 Discussion

Upright radiotherapy has the potential to reduce costs, and increase accessibility(1). In this study we explored several upright positioning options for breast radiotherapy. Beam access and treatment field lengths were assessed using pseudo-CTs generated from surface scans. Comfort and ISF size were assessed for the different positions, both with and without the Chabner XRT Bra.

In the first part of this study we investigated beam access for photon treatments. As the breasts lie more medially for upright positioning (compared to supine positioning, where they fall laterally under gravity), the question of whether upright positioning may lead to unwanted doses to the contralateral breast for photon treatment plans has thus far been unanswered (Boisbouvier & Underwood et al). The present work is the first investigation into photon beam access for upright breast radiotherapy. Mirroring the findings of Boisbouvier & Underwood et al, bras designed to be used as radiotherapy immobilisation accessories were shown to be effective in minimising ISF measurements for upright treatment positions. However, by bringing the breasts closer together, the bra increases the complexity of photon treatment planning, due to the greater risk of contralateral breast clipping. Different photon beam angles were found to be optimal for different superior-inferior sections of the breast. To address the challenge of contralateral breast clipping, a sliding window, slow-rotating gantry, and sliding jaw approach is proposed. This method preserved an approximate half-beam block geometry and allowed for sufficient target coverage in all but one case. The exception involved a participant who was unable to raise their arms high enough due to general frailty, limiting beam access to the upper breast. Notably, the uppermost 2cm of the target volume presented the most frequent beam accessibility issues, primarily due to arm clipping. CTV based planning, where a target is drawn on a CT scan and the treatment is designed to that, has been shown to have an impact on the treatment size. This could impact the field height. In this study, surface scanning was used to quantify the angles and the availability of beam angles, but in a clinical environment, trained RTTs would be able to assess the likelihood of clipping or limitations on beam angle visually, prior to the treatment planning CT.

While this paper focussed on beam arrangements around the LPO and RAO quadrants of the arc, it is worth considering the potential role of partial-arc VMAT. In this work, we presented a solution to tangential VMAT, but didn’t consider larger arcs that cross the patient’s midline. While this wasn’t directly assessed in this study, this could be feasible in the upright position with the right optimisation settings, such as the lateral breast and arm protection. This study focussed on whole-breast radiotherapy, which involves larger field sizes and presents greater challenges in terms of arm clipping, and dose to the ISF. However, partial breast radiotherapy is increasingly being used (16) and would reduce the complexity associated with larger treatment fields and arm/breast clipping. This could potentially expand beam access options, even allowing the use of the arms-by-side position, depending on target location.

In the second part of this study we examined the impact of the Chabner XRT Bra on the size of the ISF. The bra always reduced the ISF, although the magnitude of reduction varied. While larger bra sizes were generally associated with greater ISF reduction, the relationship was not strong. It is likely that the pendulous nature of the tissue (rather than the bra size) is a more significant indicator of whether the bra will prove beneficial in terms of reducing the ISF and the likelihood of skin toxicity. Arms-up positioning also reduced the ISF, although not to the same extent as the Chabner XRT Bra. The arms-by-sides, setup without the Chabner XRT Bra resulted in the largest mean ISF. Complete elimination of the ISF occurred in 38 out 61 cases (~62%) which is comparable with the results from Boisbouvier & Underwood et al, who reported ISF eliminated in 4 out of 7 cases (~57%) with the Chabner XRT Bra (12). In both studies, recruitment prioritised participants with large cup sizes.

The results presented here demonstrate that use of the Chabner XRT Bra has great potential to reduce the field length of the treated area, for upright positioning. In each position, the Chabner XRT Bra reduced the required superior-inferior length of the field required to cover the breast, by a mean value of 2.5cm. This reduction would benefit both proton and photon treatments. In the photon therapy, it should help reduce high-dose clipping of the ipsilateral lung. For protons, lung dose in the range-uncertainty region should also be reduced. There may also be value in exploring a single-cup design, allowing the contralateral breast to rest naturally under gravity. However, evaluating this concept is beyond the scope of the current study, and such a design may present challenges related to stability/reproducibility.

In the third part of the study, participants’ “unaided reproducibility” was used as a metric for ease of setup. In a clinical environment, radiation therapists would spend time matching the daily set-up to the one used during the patient’s simulation CT. Overall, the upright patient positioning system - used together with arm supports and a vacuum-formed cushion over the seat showed - permitted good unaided reproducibility. Participants could typically reposition themselves with sub-centimetre accuracy, without any assistance from the study team. Patients undergoing breast radiotherapy in particular often experience a lack of empowerment and dignity, which can be worsened if substantial radiographer manipulation is required to reposition the breast for treatment (17): good initial self-positioning could help to reduce these feelings. The data suggest that when the Chabner XRT Bra was taken on and off, the reproducibility of nipple positioning was lower than that of anatomical landmarks located outside of the Chabner XRT Bra. This could result in additional setup time; however, for this participant cohort, the anatomical benefits provided by the Chabner XRT Bra would likely justify any extra set-up time required. A one-arm-up, one arm down setup was also considered, which may ease the positioning burden for frail patients or those with pre-existing mobility issues in their contralateral arm. While this setup resulted in slightly worse unaided reproducibility, it is likely that all reproducibilities reported here could be improved via surface-guided radiotherapy (SGRT), other camera-based setup aids, or simpler radiographer interventions. An arms-back supine photon treatment was recently reported for a patient suffering from shoulder joint problems who was unable to keep their left armed raised (18).

Finally, we examined comfort, an important but often overlooked factor in radiotherapy. In this study, most healthy participants responded positively to both the Chabner XRT Bra and the upright treatment positions. For the positioning system, only one of 21 participants found the arms up position to be uncomfortable, and one found the shin rest uncomfortable (mean age 58.8). While the authors did not propose a specific reason, one study evaluated radiotherapy completion rates among breast cancer patients: it found that 87% of patients completed treatment, and among the 13% who did not, a small but statistically significant increase in recurrence risk was observed (19). It is reasonable to consider that improving the patient experience, including comfort, could help improve treatment adherence. Comfort has also been identified as an intervention to help patients sustain a stable position (20). While discussing comfort, it is also important to note that comfort was not benchmarked against a standard supine treatment in this healthy volunteer study. However, such comparisons have been made in previous studies involving radiotherapy patients, such as that by Boisbouvier et al (12). Dignity is another often overlooked factor in breast radiotherapy. In a study by Probst et al, 70% of patients reported feeling self-conscious, with 86% indicated a preference for wearing a gown during treatment (17). In the present study, the majority of participants indicated that they would prefer to be treated with the Chabner XRT Bra than without, while the remainder responded neutrally.

Surprisingly, most participants in this cohort preferred the arms-up position over placing their arms by their sides, or behind their back. The arms-up supports used were custom designed for upright radiotherapy: prior to this study their design was iterated based on multiple rounds of feedback from former patients, physiotherapists and a range clinical staff. Only one participant found the arms up position uncomfortable. As all participants were healthy volunteers, it is likely that issues with the arms-up position would be more prevalent among patients who have undergone breast cancer. Additionally, the backrest used in this study for the arms-back-and-behind position was an early-stage prototype. Its comfort could likely be improved through further human factors analyses and design iterations. For protons, even arms by side upright positioning should be feasible in many cases, from a treatment planning perspective (15). While arms by side positioning is incompatible with photon breast treatments, this study demonstrated that arms back positioning could serve as an alternative option for certain patients, particularly those unable to raise their arms sufficiently high for an upright arms-up set-up.

Ultimately, while this study addresses many questions surrounding upright radiotherapy of the breast, several important questions remain. The direct application of supine field borders to upright radiotherapy requires further investigation. The external anatomical markers used in this study provide a reasonable starting point for assessing field borders and beam access. However, breast tissue sits quite differently upright, compared to supine. CTVs and margins derived from internal anatomical will offer a more accurate indication of the field borders required. The present research should be followed by studies of internal anatomy (using MRI or CT) and full treatment planning studies.

This study is a major step towards developing a protocol for delivering radiotherapy to the breast in the upright position. From the perspectives of beam access and comfort, the arms-up position appears to offer the best overall solution for patients. However, for those who struggle with this position, the arms-back position often provides a viable alternative.

## 5 Conclusion

For the first time, this study demonstrates that upright positioning could be a feasible option for breast radiotherapy. This is an important finding, given the clear cost-saving potential of upright radiotherapy and the strong global need to improve access to breast cancer treatment. This study shows that upright set-ups can also often offer flexibility in arm positioning, allowing for an arms-down-and-behind configuration for patients who are unable to raise their arms up above their heads. While the external breast contour provided a useful initial indicator of the treatment area, its limitations are acknowledged. Further studies using MRI and CT are needed to fully assess internal anatomy, the feasibility of the internal mammary node irradiation and doses to organs at risk (OARs).

## Data Availability

The raw data supporting the conclusions of this article will be made available by the authors, without undue reservation.
