# Supplementary material for: Upright radiotherapy for breast cancer: a pre-clinical study considering photon and proton beam access, plus arm positioning
Source: Front Oncol. 2025 Dec 10;15:1668109. doi: 10.3389/fonc.2025.1668109 (PMC12727594; doi:10.3389/fonc.2025.1668109)
Supplement: Supplementary file 1 [file DataSheet1.docx]

**Supplementary information**

1. **Marker position**


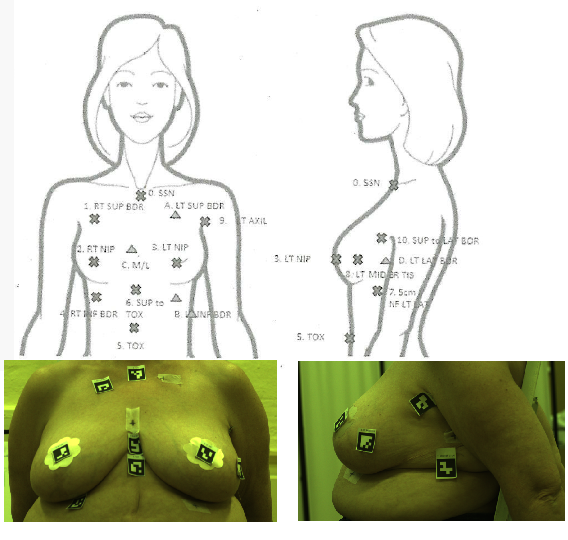


Supplementary figure 1: top - positions of the aruco markers. Botton – example photographs of the markers taped in position.

1. Method for treatment planning

In a clinical setting, this approach would of course be complemented with beam modulation (e.g. using a field-in-field or wedge approach) to create a uniform dose distribution. This treatment planning element of this study considered beam access and field length only, so modulation was not considered. This approach for field angle analysis could also be used to examine the feasibility of short arc VMAT. We will present data about acceptable angles of entry and number of angles


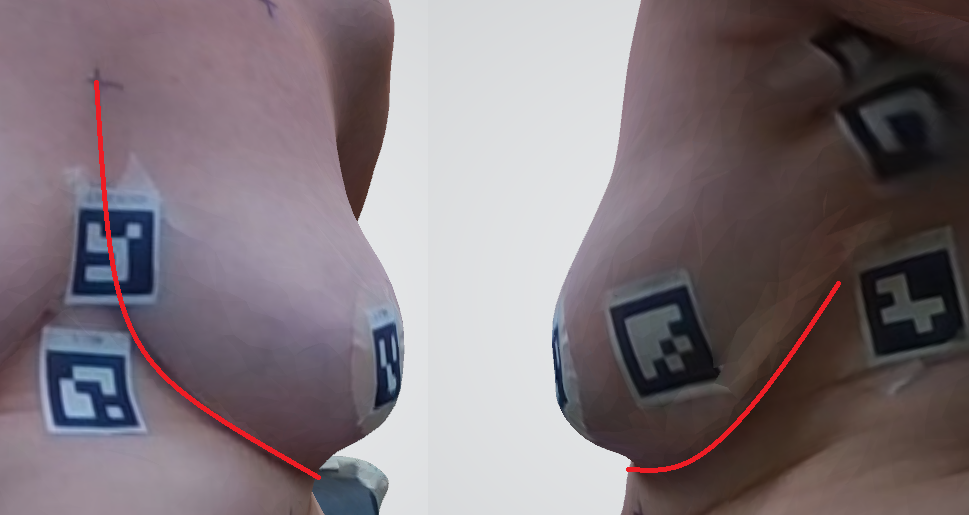
A single straight line was used for the superior border of the breast contour. The line was placed 2cm below the sternal notch. Historically, the sternal notch has often been used as a reference point, although the reported distance of the target edge from the sternal notch varies somewhat in the literature. As the external contour of the breast became apparent, the central border followed the curvature of the breast tissue, as highlighted in supplementary figure 2. On the side of the tissue, preference was given to the curvature of the breast tissue. The mid-axillary line was used to define the field edge in the superior part of the tissue, where the breast tissue curvature wasn’t clear. A straight line was used to join the left-side field border with the midline border. The inferior border was at the most inferior part of the breast tissue as viewed in the TPS using the shape of the breast. The curvature of the breast and the equivalent field border are seen in Supplementary figure 2. This becomes our estimation of the CTV for beam angle estimation. Supplementary figure 3 shows the conversion of the 3d scan to the dicom plan in Raystation.

Supplementary figure 2: Highlighting the curvature of the breast for the CTV

Supplementary
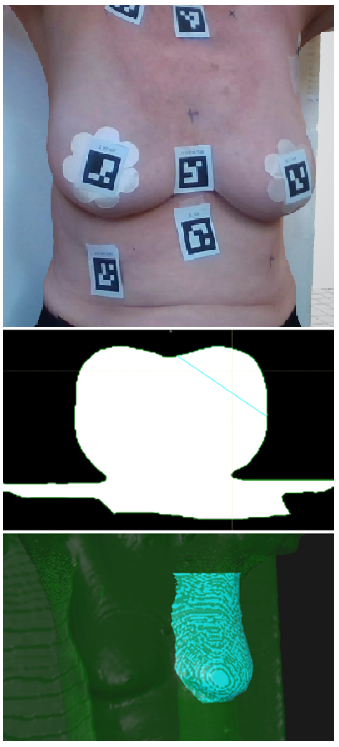
figure 3: Top: 3D surface scan, middle: single slice from dicom converted scan, bottom: 3d render of dicom conversion with an overlay of breast contour


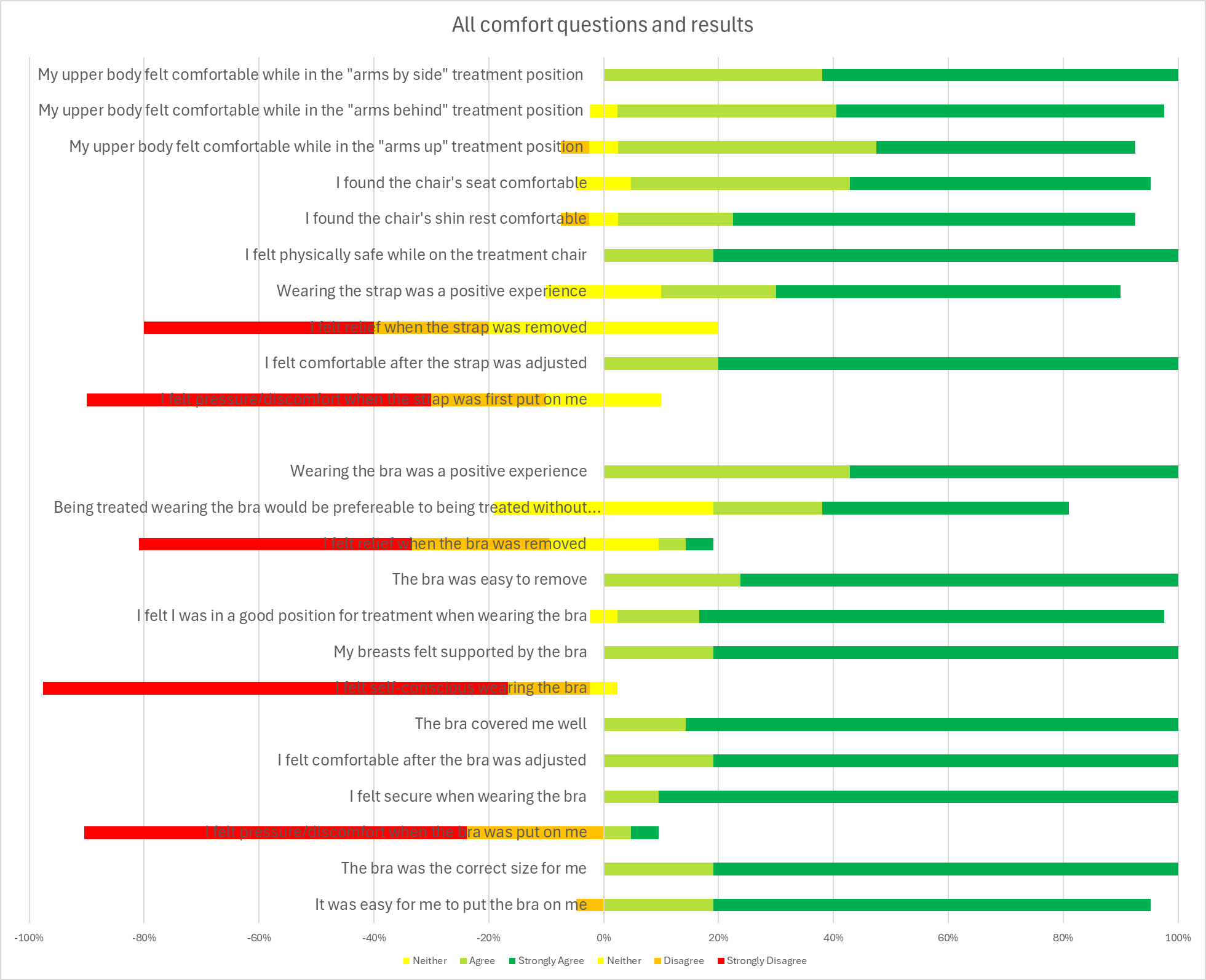


Supplementary figure 4: All comfort questions and results
